# Heterogeneity and distribution characteristics of tertiary lymphoid structures predict prognostic outcome in esophageal squamous cell carcinoma

**DOI:** 10.3389/fimmu.2025.1606499

**Published:** 2025-08-08

**Authors:** Chengjuan Zhang, Ruihua Bai, Yanping Hu, Tao Wang, Bin Ma, Junxia Zhang, Jing Yuan, Xiance Tang, He Zhang, Tingjie Wang, Yuxi Chang, Qingxin Xia, Bing Wei

**Affiliations:** ^1^ Center of Bio-Repository, The Affiliated Cancer Hospital of Zhengzhou University & Henan Cancer Hospital, Zhengzhou, China; ^2^ Department of Pathology, The Affiliated Cancer Hospital of Zhengzhou University & Henan Cancer Hospital, Zhengzhou, China; ^3^ Department of Molecular Pathology, The Affiliated Cancer Hospital of Zhengzhou University& Henan Cancer Hospital, Zhengzhou, China; ^4^ The Kids Research Institute Australia, School of Medicine the University of Western Australia, Nedlands, WA, Australia; ^5^ School of Medical, Molecular and Forensic Sciences, Murdoch University, Murdoch, WA, Australia; ^6^ Academy of Chinese Medical Sciences, Henan University of Chinese Medicine, Zhengzhou, China

**Keywords:** esophageal cancer, tertiary lymphoid structures, heterogeneity, prognosis their heterogeneity in esophageal squamous univariate analysis revealed that T stage, N stage, TG, TR, nr

## Abstract

**Objective:**

Tertiary Lymphoid Structures (TLSs) are ectopic lymphoid aggregates that form within the tumor microenvironment (TME) and are increasingly recognized as potential prognostic biomarkers in various cancers. However, the spatial heterogeneity and prognostic value of TLSs in esophageal squamous cell carcinoma (ESCC) remain poorly defined. This study aimed to characterize the spatial distribution patterns of TLSs and tumor-infiltrating lymphocytes (TILs), and to establish a refined prognostic model for ESCC patients in both surgery-only and neoadjuvant therapy cohorts.

**Methods:**

The TLSs were quantified through microscopic evaluation and digital slide analysis and correlated with prognosis by Cox regression and Kaplan-Meier analyses. The heterogeneity and clinical prognostic value of TLSs were explored by analyzing their distribution, density, and maximum diameter in different regions of ESCC patients.

**Results:**

TLSs showed spatial distribution heterogeneity in the tumor area, adjacent area, and marginal area, with consistent differences observed across different paraffin blocks. The distribution of iTIL and sTIL also exhibited certain spatial heterogeneity. In the surgical cohort (n = 117), the median Overall Survival (OS) and Disease-Free Survival (DFS) were 33 months and 15 months, respectively. Univariate analyses showed that TLS presence in tumor (TG), TLS-rich regions (TR), TLS ratio in normal regions (NR), tumor-stroma ratio (TSR), and both iTIL and sTIL levels were significantly associated with OS (*p* < 0.05). Multivariate analysis confirmed N stage, TG, TR, TLS abundance in adjacent regions (NA), and TLS density in tumor (NT), along with TSR, iTIL, and sTIL, as independent predictors of prognosis (*p* < 0.05). High TLS presence in tumor regions (TG-high) was associated with significantly improved OS (log-rank *p* = 0.026).

**Conclusion:**

This study demonstrates that TLSs and TILs in ESCC are not only prognostically relevant but also spatially heterogeneous. The refined spatial immune profiling across multiple tumor regions improves prognostic stratification and may inform personalized treatment planning in ESCC.

## Introduction

Esophageal cancer is the sixth leading cause of cancer-related deaths worldwide, with an overall 5-year survival rate of approximately 10% and a 5-year survival rate of 15–40% after resection; approximately 70% of esophageal cancer cases occur in China (1). Esophageal cancer includes esophageal adenocarcinoma and esophageal squamous cell carcinoma (ESCC) (2). ESCC accounts for more than 90% of all esophageal cancers in China (3). Research has shown that environmental exposure, lifestyle, and genetic characteristics are the factors responsible for the high incidence of ESCC in China, with environmental factors being the main driving factors (4).

Although with the advent of neoadjuvant concurrent chemoradiotherapy (CCRT), the management, diagnosis, surgical prognosis, and treatment of esophageal cancer patients have improved in recent decades, the overall outcome is still poor, and new treatment methods often result in increased toxicity and side effects (5). Surgical resection remains the mainstream treatment method for early esophageal cancer. However, most patients with esophageal cancer are at a locally advanced stage at the time of diagnosis, and the therapeutic effect of simple surgery is limited (6). Although CCRT has improved the survival rates of patients, nearly half of them still experience local recurrence or distant metastasis after surgery; additionally, CCRT causes different degrees of toxicity and side effects in patients and increases their pain. Therefore, there is an urgent need to explore new and effective diagnostic and treatment methods to improve the survival rates of patients (7).

Immunotherapy is poised to play an increasingly pivotal role in the management of esophageal cancer. Tumor-infiltrating T cells are central to the immune response within the tumor microenvironment, a critical determinant of immunotherapy’s effectiveness. Extensive research has elucidated the role of cytotoxic T cells in this context. Concurrently, investigations are exploring the dichotomous impact of B cells, assessing their potential as either tumor promoters or anti-tumor agents. Tertiary lymphoid structures (TLSs) are ectopic lymphoid tissue that are temporarily formed in the affected area, not the secondary lymphoid organs, and are mainly composed of follicular dendritic cells, B cell regions, T cell regions, and high endothelial venules (HEVs) (8). It has recently been reported that TLSs are found in the microenvironment of tumors (9).

There are reports that the presence of TLSs around gastric cancer is associated with a good prognosis, that increased granzyme B and perforin expression is observed around TLSs, and that memory T cells are present around TLSs (10). Zhao et al. identified TLS-rich as a prognostic factor for superficial esophageal cancer (11). Additionally, Ruffin et al. emphasized the significance of B-cell function in forming TLSs and internal Germinal Center (GC) in head and neck cancers with histological resemblance to ESCC (12). Meanwhile, Esophageal cancer is a unique and complex heterogeneous malignancy, with substantial tumor heterogeneity (13), and TLS at different sites also showed significant heterogeneity, which determines tumor immunity and prospects for clinical application (14). There is often more than one tumor wax block in a diseased individual. What is the consistency of TLS results for different tumor wax blocks in the same individual? At present, the heterogeneity of TLS in ESCC and its relationship with prognosis are unclear.

This study aimed to address the heterogeneity of TLS and its clinical prognostic value through the presence, density and maximum diameter in different sites of ESCC patients. Meanwhile, a convenient method for pathologists to evaluate TLS was established to provide more clues for judging the prognosis and clinical transformation of patients with ESCC.

## Materials and methods

### Case selection and study design

**Figure 1:**
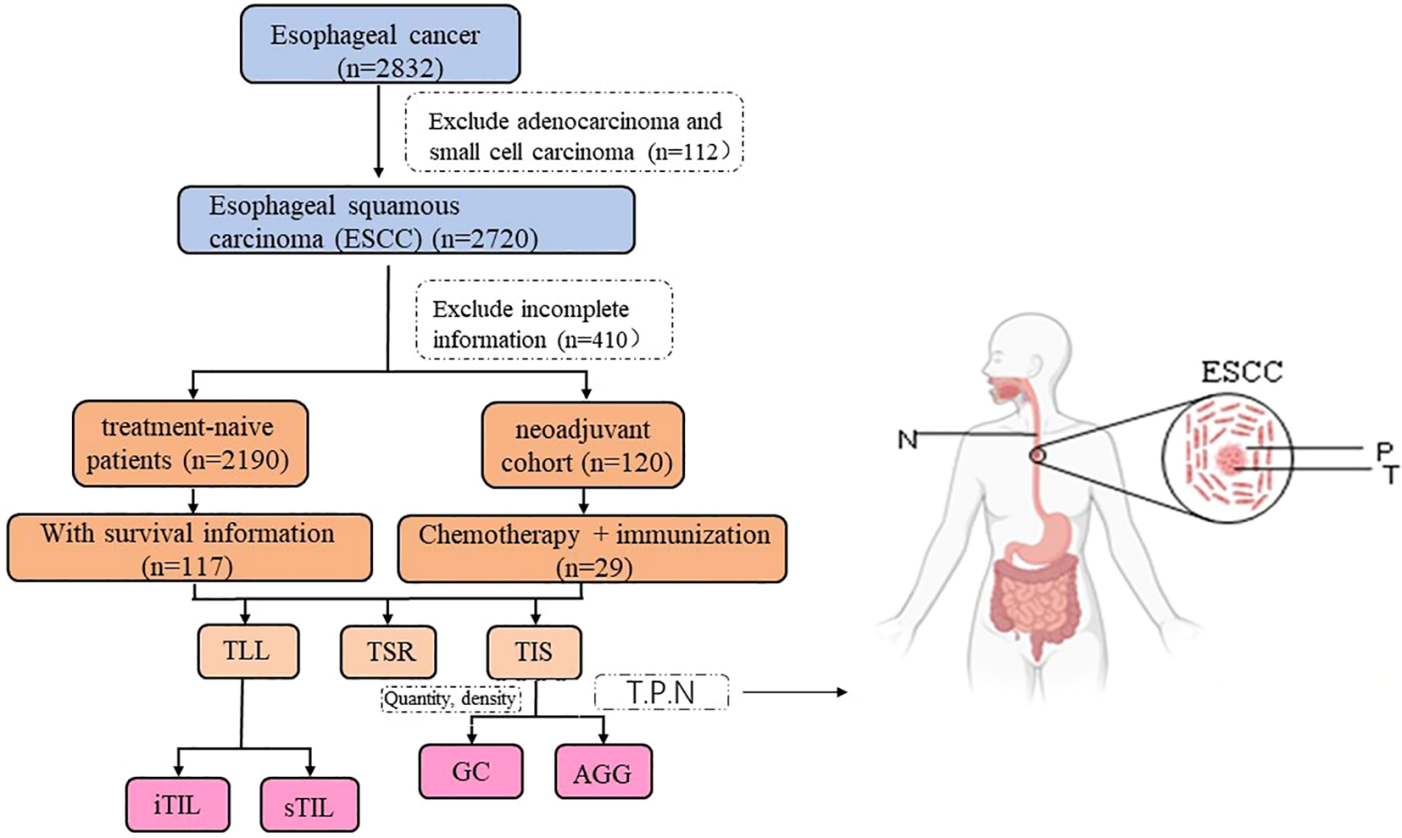
Cases selection and study design. (T, P, N stands for Tumor, Peritumor, and Normal margin, respectively). The surgery cohort refers to patients who underwent direct surgery without prior neoadjuvant treatment. The neoadjuvant cohort includes patients who received immunochemotherapy before surgery.

By screening the pathological information of esophageal cancer patients hospitalized at Henan Cancer Hospital (China) from January 2015 to December 2022, and combining follow-up data, and the ESCC cohort was analyzed for heterogeneity (n=117) and prognostic assessment (n=112). The case screening process and research design are shown in **Figure 1**. This study was conducted in accordance with the Declaration of Helsinki and received approval from the Ethics Committee of Henan Cancer Hospital (Ethics Approval No: 2021-KY-0092–001). Written informed consent was obtained from all participants involved in the research.

By consulting the medical records, we confirmed the clinical and pathological data of qualified cases (including the age of first diagnosis, gender, treatment plan and therapeutic effect, etc.) and made statistical analysis. The patients were mainly divided into two cohorts: surgery group and neoadjuvant treatment group. Surgery cohort refers to cases that do not receive neoadjuvant therapy. The neoadjuvant cohort refers to those who received chemotherapy drugs and immunological drugs before surgery.

### Hematoxylin and eosin staining

The paraffin-embedded ESCC tissue specimens were cut into 5 μm thick continuous sections and baked in the oven for 2 h at 65°C. The tissue sections were successively transferred to xylene and alcohol for dewaxing to water; Hematoxylin solution was stained for 5 min and differentiated into 1% hydrochloric acid and alcohol. Water back blue after eosin staining; Transfer to different concentrations of alcohol (75%, 85%, 95%, 100%) and xylene in order to dehydrate transparent, neutral gum tablets. The results of HE staining were observed by optical microscope.

### Quantification and detection of TLSs

Two pathologists who were unaware of the patient’s clinical information and prognosis observed the HE sections with a microscope to assess whether TLS was present in the tumor, and counts the number of TLSs. The number of TLSs in each section was also recorded for peritumoral (5 mm area around tumor tissue) and incisal margin. Qupath software was used to scan HE slices, and TLSs (presence/quantity) was determined twice according to the preliminary screening results. For TLS positive cases, the diameter of the largest TLS and the tumor area and peri-tumor area were measured. Tumor area (T area), incisal margin area (N area), peritumoral area (P area). Here, TG, TA, and TT represent the presence of GC, Aggregates (AGG) and total TLS in the tumor region, respectively. PG, PA, and PT represent the presence of GC, AGG, and total TLS in the peritumoral area, respectively. NG, NA, and NT represent the presence of GC, AGG, and total TLS in the incisal margin area, respectively. We analyzed the TLS distribution and density of all tumor wax blocks (four-eight) in each case, and analyzed the spatial heterogeneity among different sections.

### TSR/TIL definition and detection

Tumor infiltrating lymphocytes (TILs) include intratumoral tumor infiltrating lymphocytes (iTIL) and stromal tumor infiltrating lymphocytes (sTIL) in the stroma that are present in the tumor’s cancer nest. The clinical information was unknown by two independent pathologists in advance. The tumor-stroma ratio (TSR) 50% was taken as the truncation value. TSR<50% and TSR≥50% were defined as the interstitial rich group and the interstitial sparse group, respectively, so as to compare the prognosis difference between the two groups.

### Multiplex immunofluorescence staining

Multiplex immunofluorescence staining was performed on formalin-fixed, FFPE tissue sections to assess TLS-related immune cell distribution in both tumor and peritumoral areas. Briefly, 4-μm-thick sections were deparaffinized, rehydrated, and subjected to antigen retrieval using citrate buffer (pH 6.0) in a pressure cooker. Endogenous peroxidase activity was blocked with 3% hydrogen peroxide, followed by incubation in 5% BSA blocking buffer. Primary antibodies were applied sequentially using tyramide signal amplification (TSA), including CD3 (FITC, 520 nm), CD8 (Texas Red, 620 nm), CD20 (Cy5, 690 nm), and pan-cytokeratin (TRITC, 570 nm), with DAPI used for nuclear counterstaining. Each antibody was followed by HRP-conjugated secondary antibody and TSA fluorophore, with microwave treatment for antibody stripping between rounds. Stained slides were scanned using a fluorescence microscope with appropriate filters, and images were analyzed using image processing software.

### Statistical analysis

SPSS 25.0 software was used for statistical analysis. Continuous variables are presented as mean ± standard error (SE) or median (interquartile range, IQR), and categorical variables are expressed as frequency (percentage). Univariate and multivariate analyses were performed using Cox proportional hazards regression models, with hazard ratios (HRs) and corresponding 95% confidence intervals (CIs) calculated. The data were compared between groups using the χ 2 test and paired t- test, as appropriate. Survival curves were generated by the Kaplan-Meier method and compared using log-rank tests. All statistical tests were two-sided, with *p* values <0.05 considered statistically significant.

## Results

### Clinicopathological characteristics of two tumor research cohorts

In the surgery cohort, the median overall survival (OS) and disease-free survival (DFS) were 33 months and 15 months, respectively (n=112). Tumor staging analysis revealed that most patients presented with advanced disease: T3 (51.8%) was the most common, followed by T2 (36.6%), T1 (9.8%), and T4 (1.8%). In terms of lymph node involvement, 48.2% were classified as N0, 33.9% as N1, and 17.9% as N2 (**Table 1**). In the neoadjuvant cohort, the number of cases with Tumor regression grade(TRG)classification 0, 1, 2, 3 were 9, 6, 11, 3, respectively. More than two-thirds of patients were men (72.3% in the surgery cohort and 75.8% in the validation neoadjuvant cohort). The median age was 66 years (range: 50–79 years) in the surgery cohort and 63 years (range: 47–74 years) in the neoadjuvant cohort. Univariate analysis identified several factors significantly associated with overall survival (OS), including T stage (p = 0.027), N stage (p = 0.002), tumor grade (TG, p = 0.026), tumor regression (TR, p = 0.008), necrosis ratio (NR, p = 0.043), tumor-stroma ratio (TSR, p = 0.006), intratumoral tumor-infiltrating lymphocytes (iTIL, *p* < 0.001), and stromal TILs (sTIL, *p* < 0.001). However, in the multivariate analysis, T stage lost its statistical significance (p = 0.537), suggesting it may be confounded by other variables. Independent predictors of OS included N stage, TG, TR, necrosis area (NA), necrosis grade (NG), necrosis type (NT), TSR, iTIL, and sTIL (**Table 1**). Additionally, the analysis results for DFS showed that in the univariate analysis, T stage (p = 0.027), PA (p = 0.024), PG (p = 0.047), and PT (p = 0.006) were all significantly associated with DFS. In the multivariate analysis, only PA (p = 0.044) was identified as an independent predictive factor (**Table 2**). The analysis results indicate that the factors influencing OS and DFS are not entirely the same.

**Table 1:** Univariable and Multivariate Cox analysis of OS for 112 ESCC patients.

Characteristic		All (%)	HR (univariable)	HR (multivariable)
Gender	Female	31 (27.7)	–	–
	Male	81 (72.3)	1.00 (0.64-1.58, p=0.987)	0.81 (0.37-1.73, p=0.580)
Age	Mean (SD)	66.8 (7.3)	1.00 (0.97-1.03, p=0.841)	1.02 (0.99-1.06, p=0.186)
T	T1	11 (9.8)	–	–
	T2	41 (36.6)	2.22 (0.93-5.29, p=0.072)	1.47 (0.52-4.18, p=0.465)
	T3	58 (51.8)	2.59 (1.11-6.05, **p=0.027**)	1.38 (0.50-3.83, p=0.537)
	T4	2 (1.8)	2.60 (0.52-12.95, p=0.243)	3.34 (0.46-24.43, p=0.234)
N	N0	54 (48.2)	–	–
	N1	38 (33.9)	2.05 (1.29-3.26, **p=0.002**)	1.83 (1.02-3.27, **p=0.043**)
	N2	20 (17.9)	2.18 (1.25-3.81, **p=0.006**)	2.60 (1.28-5.30, **p=0.008**)
Smoking	No	57 (50.9)	–	–
	Yes	55 (49.1)	1.29 (0.86-1.93, p=0.227)	1.70 (0.82-3.51, p=0.152)
Drinking	No	61 (54.5)	–	–
	Yes	51 (45.5)	1.15 (0.76-1.72, p=0.508)	1.71 (0.92-3.19, p=0.090)
TA	Mean (SD)	6.9 (6.7)	1.00 (0.97-1.04, p=0.802)	0.99 (0.94-1.03, p=0.561)
TG	Mean (SD)	0.8 (2.7)	0.85 (0.73-0.98, **p=0.026**)	0.72 (0.53-0.97, **p=0.029**)
TT	Mean (SD)	7.7 (7.6)	0.99 (0.96-1.02, p=0.446)	NA (NA-NA, p=NA)
TD	Mean (SD)	0.03 (0.02)	0.24 (0.00-236.95, p=0.685)	0.00 (0.00-266.28, p=0.321)
TR	Mean (SD)	0.5 (0.4)	2.09 (1.21-3.62, **p=0.008**)	2.28 (1.00-5.17, **p=0.049**)
PA	Mean (SD)	10.8 (5.4)	1.01 (0.97-1.05, p=0.768)	0.95 (0.90-1.01, p=0.098)
PG	Mean (SD)	1.9 (4.5)	0.94 (0.87-1.01, p=0.093)	1.02 (0.95-1.09, p=0.552)
PT	Mean (SD)	12.7 (7.7)	0.99 (0.96-1.01, p=0.332)	NA (NA-NA, p=NA)
PR	Mean (SD)	0.8 (0.5)	1.35 (0.97-1.86, p=0.072)	0.95 (0.49-1.81, p=0.865)
NA	Mean (SD)	3.0 (3.1)	1.01 (0.96-1.08, p=0.613)	0.00 (0.00-0.39, **p=0.026**)
NG	Mean (SD)	1.6 (2.7)	0.93 (0.85-1.01, p=0.088)	0.00 (0.00-0.37, **p=0.025**)
NT	Mean (SD)	4.5 (4.3)	0.98 (0.94-1.03, p=0.483)	2236.39 (2.39-2092947.77, **p=0.027**)
NR	Mean (SD)	0.4 (0.3)	1.95 (1.02-3.71, **p=0.043**)	0.89 (0.33-2.36, p=0.812)
ND	Mean (SD)	0.2 (0.2)	2.66 (0.77-9.22, p=0.124)	4.17 (0.68-25.58, p=0.122)
TSR	≥50	108 (96.4)	–	–
	<50	4 (3.6)	4.17 (1.50-11.61, **p=0.006**)	10.23 (2.22-47.18, **p=0.003**)
iTIL	Mean (SD)	6.4 (4.1)	1.16 (1.10-1.22, **p<0.001**)	1.14 (1.07-1.22, **p<0.001**)
sTIL	Mean (SD)	36.7 (19.1)	1.03 (1.02-1.04, **p<0.001**)	1.02 (1.01-1.04, **p=0.003**)

TSR, Tumor-Stroma Ratio; TA, Tumor Aggregate; TG, Tumor Germinal Center; TT,Total tumor (TA+TG); TD,Tumor Density; TLS, Tertiary Lymphoid Structures; TR, Tumor Diameter of TLS; PA, Peritumoral Aggregate; PG, Peritumoral Germinal Center; PT,Total Peritumoral (PA+PG); PR, Peritumoral Radius of TLS; NA, Normal Tissue Aggregate; NG, Normal Tissue Germinal Center; NT, Total Normal Tissue (NA+NG); ND, Normal Tissue Density of TLS; NR, Normal Tissue Radius of TLS; iTIL, intratumoral tumor-infiltrating lymphocytes; sTIL, stromal tumor-infiltrating lymphocytes.

Bold p-values indicate statistical significance (p < 0.05).

**Table 2:** Univariable and Multivariate Cox analysis of DFS for 112 ESCC patients.

Characteristic		All (%)	HR (univariable)	HR (multivariable)
Gender	Female	31 (27.7)	–	–
	Male	81 (72.3)	1.11 (0.47-2.62, p=0.804)	1.00 (0.20-5.07, p=0.999)
Age	Mean (SD)	66.8 (7.3)	0.97 (0.92-1.02, p=0.268)	0.97 (0.92-1.03, p=0.313)
T	T1	11 (9.8)	–	–
	T2	41 (36.6)	1.25 (0.26-5.88, p=0.781)	0.65 (0.11-3.73, p=0.624)
	T3	58 (51.8)	2.06 (0.47-9.01, p=0.339)	1.07 (0.20-5.80, p=0.941)
	T4	2 (1.8)	9.17 (1.28-65.72, p=**0.027**)	8.16 (0.50-133.04, p=0.140)
N	N0	54 (48.2)	–	–
	N1	38 (33.9)	1.10 (0.44-2.71, p=0.838)	1.00 (0.32-3.12, p=0.995)
	N2	20 (17.9)	2.40 (0.97-5.94, p=0.057)	1.72 (0.56-5.31, p=0.343)
Smoking	No	57 (50.9)	–	–
	Yes	55 (49.1)	1.74 (0.82-3.72, p=0.152)	2.51 (0.61-10.29, p=0.202)
Drinking	No	61 (54.5)	–	–
	Yes	51 (45.5)	1.10 (0.52-2.31, p=0.807)	1.66 (0.51-5.38, p=0.399)
TA	Mean (SD)	6.9 (6.7)	0.95 (0.88-1.03, p=0.188)	0.99 (0.90-1.10, p=0.909)
TG	Mean (SD)	0.8 (2.7)	0.53 (0.27-1.04, p=0.066)	0.61 (0.29-1.29, p=0.195)
TT	Mean (SD)	7.7 (7.6)	0.94 (0.87-1.01, p=0.076)	NA (NA-NA, p=NA)
TD	Mean (SD)	0.03 (0.02)	0.00 (0.00-1652.64, p=0.361)	2.87 (0.00-9923441502.05, p=0.925)
TR	Mean (SD)	0.5 (0.4)	0.59 (0.18-1.90, p=0.373)	0.94 (0.16-5.46, p=0.942)
PA	Mean (SD)	10.8 (5.4)	0.91 (0.83-0.99, **p=0.024**)	0.87 (0.76-1.00, p=**0.044**)
PG	Mean (SD)	1.9 (4.5)	0.77 (0.59-1.00, **p=0.047**)	0.84 (0.63-1.13, p=0.248)
PT	Mean (SD)	12.7 (7.7)	0.89 (0.82-0.97, **p=0.006**)	NA (NA-NA, p=NA)
PR	Mean (SD)	0.8 (0.5)	0.66 (0.27-1.58, p=0.347)	1.68 (0.47-6.04, p=0.428)
NA	Mean (SD)	3.0 (3.1)	1.04 (0.94-1.16, p=0.449)	57.89 (0.00-Inf, p=1.000)
NG	Mean (SD)	1.6 (2.7)	1.00 (0.87-1.16, p=0.948)	52.77 (0.00-Inf, p=1.000)
NT	Mean (SD)	4.5 (4.3)	1.02 (0.95-1.10, p=0.580)	0.02 (0.00-Inf, p=1.000)
NR	Mean (SD)	0.4 (0.3)	1.01 (0.30-3.44, p=0.985)	2.66 (0.39-18.16, p=0.318)
ND	Mean (SD)	0.2 (0.2)	1.64 (0.16-16.54, p=0.673)	0.99 (0.02-49.40, p=0.994)
TSR	≥50	108 (96.4)	–	–
	<50	4 (3.6)	0.00 (0.00-Inf, p=0.998)	0.00 (0.00-Inf, p=0.998)
iTIL	Mean (SD)	6.4 (4.1)	0.98 (0.88-1.08, p=0.677)	0.95 (0.82-1.10, p=0.463)
sTIL	Mean (SD)	36.7 (19.1)	0.98 (0.96-1.00, p=0.091)	0.99 (0.95-1.02, p=0.379)

TSR, Tumor-Stroma Ratio; TA, Tumor Aggregate; TG, Tumor Germinal Center; TT,Total tumor (TA+TG); TD,Tumor Density; TLS, Tertiary Lymphoid Structures; TR, Tumor Diameter of TLS; PA, Peritumoral Aggregate; PG, Peritumoral Germinal Center; PT,Total Peritumoral (PA+PG); PR, Peritumoral Radius of TLS; NA, Normal Tissue Aggregate; NG, Normal Tissue Germinal Center; NT, Total Normal Tissue (NA+NG); ND, Normal Tissue Density of TLS; NR, Normal Tissue Radius of TLS; iTIL, intratumoral tumor-infiltrating lymphocytes; sTIL, stromal tumor-infiltrating lymphocytes.

Bold p-values indicate statistical significance (p < 0.05).

These results highlight that while several pathological and immunological features are associated with survival outcomes, the factors independently predicting OS and DFS differ. OS appears to be more strongly influenced by tumor biology and immune microenvironment, whereas DFS is more closely linked to features of perineural invasion.

### Spatial distribution characteristics of TLSs in esophageal cancer

**Figure 2:**
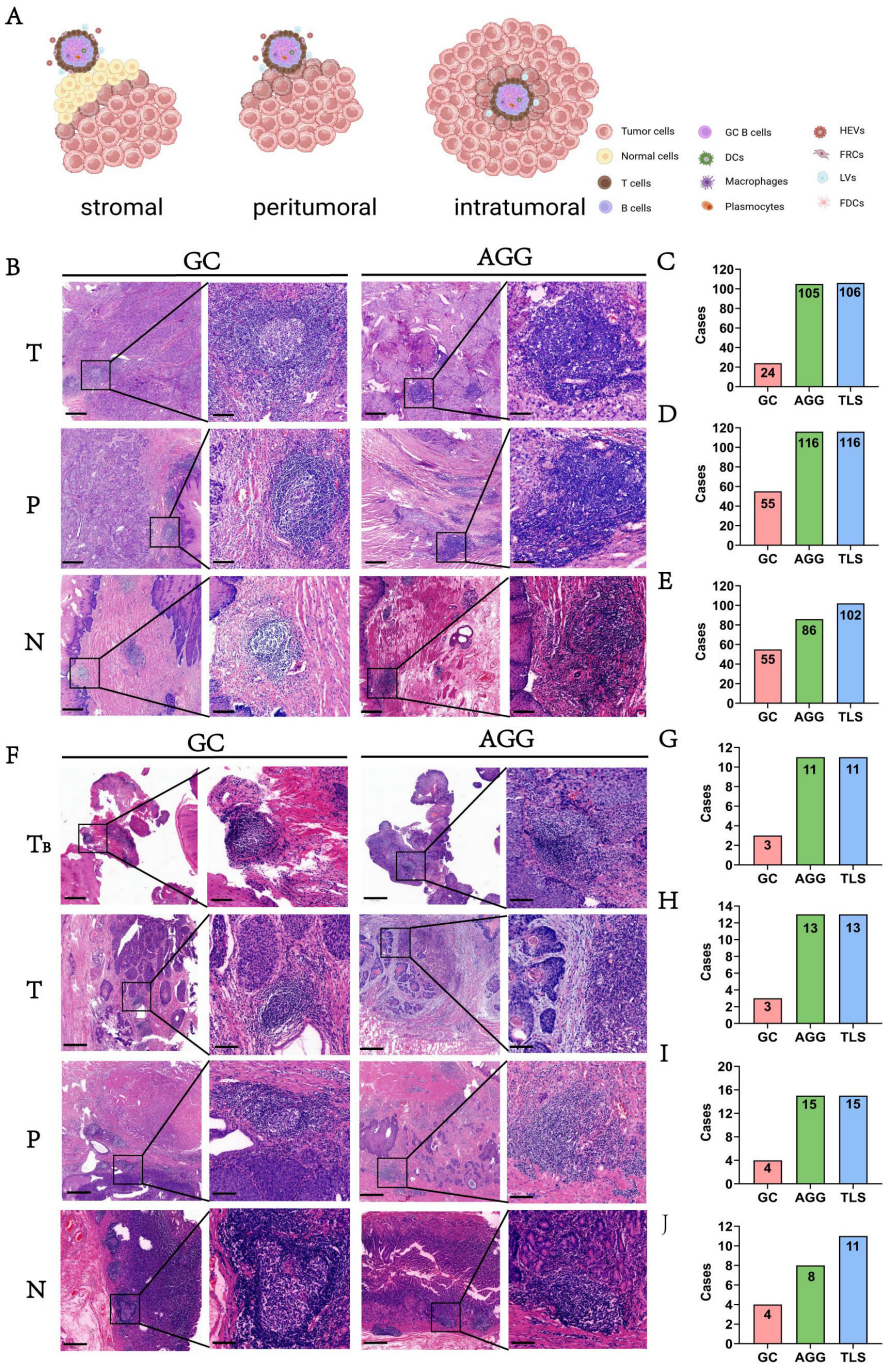
The morphology and distribution of GC and AGG expression in different tissue regions. **(A)** Schematic illustration showing the spatial distribution of TLS within intra-tumoral, peri-tumoral margin, and extra-tumoral (stromal) regions. **(B)** The HE morphology of GC and AGG in the ESCC surgery cohort. **(C-E)** Represent the distribution of GC, AGG, and TLS in different regions of T, P and N in the ESCC surgery cohort. **(F)** The HE morphology of GC and AGG in the ESCC neoadjuvant cohort. T_B_ represents tumor biopsy tissue before neoadjuvant therapy. **(G-J)** Represent the distribution of GC, AGG, and TLS in different regions of T, P and N in the ESCC neoadjuvant cohort.

The spatial distribution of tertiary lymphoid structures (TLSs) in esophageal cancer varied across tumor (T), peritumoral (P), and nodal (N) regions. As illustrated in **Figure 2A**, TLS were observed in distinct spatial compartments, including intra-tumoral, peri-tumoral margin, and extra-tumoral (stromal) regions. Representative histological features of TLSs, including GCs and AGGs, are shown in **Figure 2A**. In the surgery cohort, varying numbers of GC and AGG were observed in the T, P, N regions. The HE morphology of TLS is illustrated in **Figure 2B**. Within the tumor region, 106 patients (90.6%) were TLS-positive, among which 24 (20.5%) and 105 (89.7%) exhibited GC and AGG, respectively (**Figure 2C**). In the peritumoral region, 116 cases (99.1%) were TLS-positive, with 55 cases (47.0%) showing GC structures (**Figure 2D**). At the tumor margin, the numbers of GC-positive, AGG-positive, and TLS-positive cases were 55 (47.0%), 86 (73.5%), and 102 (87.2%), respectively (**Figure 2E**). TLS distribution was also assessed before and after neoadjuvant treatment, with results summarized in **Figures 2F–J**. In the T regions before and after treatment, GC structures were identified in 3 cases each, and AGGs in 11 and 13 cases, respectively. In the P region, GCs and AGGs were found in 4 and 15 cases, respectively. In the N region, 4 patients had GCs, and 8 had AGGs.

These results demonstrate that AGGs are consistently more frequent than GCs across all spatial regions and treatment phases. Furthermore, TLSs remain prevalent even after neoadjuvant therapy, indicating a potentially persistent immune response within the tumor microenvironment.

### TLSs exhibit spatial distribution heterogeneity

**Figure 3:**
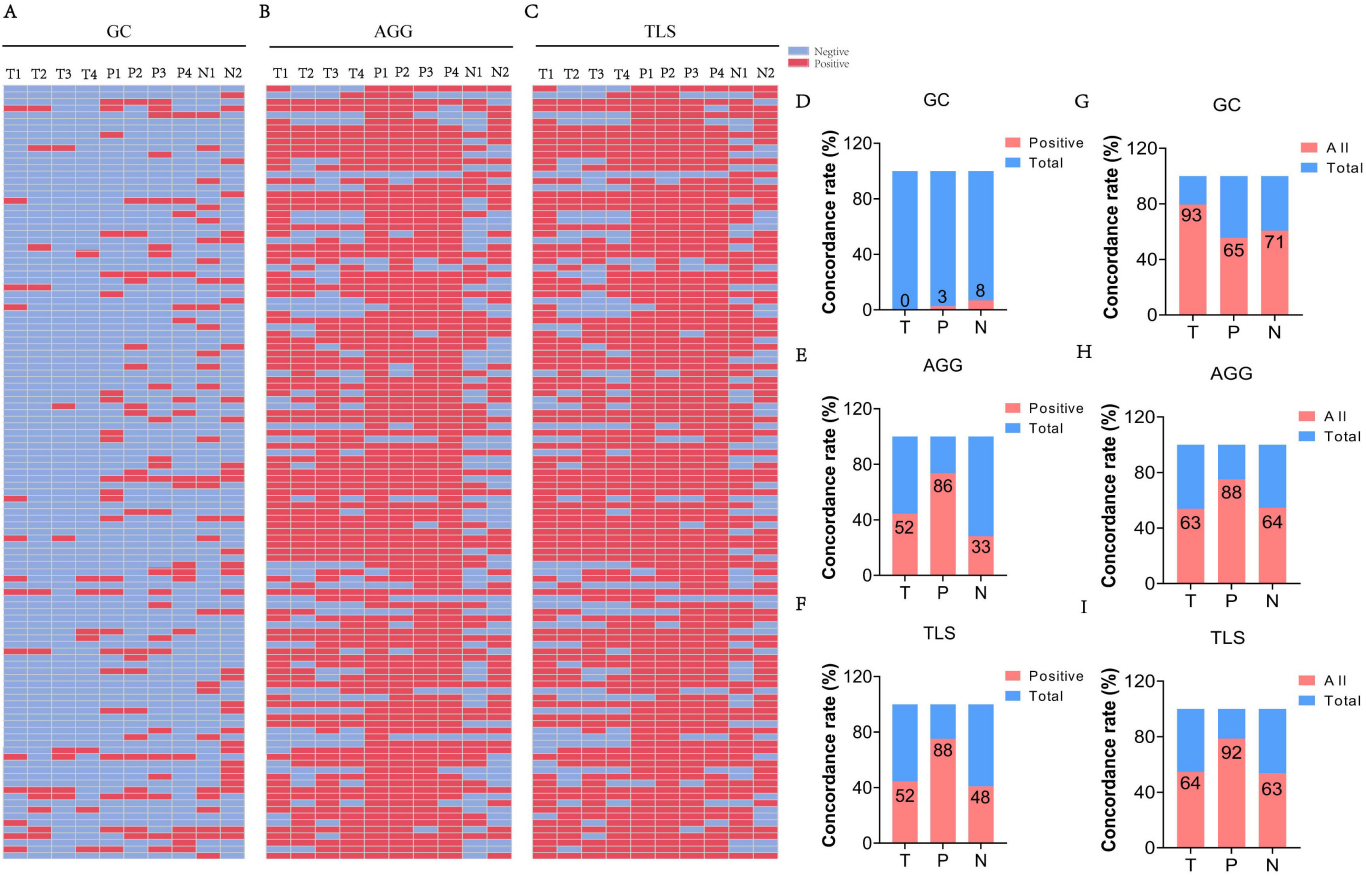
The heterogeneity of TLS expression in different regions (T, P, N) of ESCC cases. **(A-C)** Expression of GC, AGG, and TLS in different paraffin blocks of each ESCC case. (T1–4 represent different tumor paraffin blocks from the same case, respectively, while P1–4 denote the adjacent peritumoral tissues corresponding to each tumor block. N1–2 represent the two surgical margin tissues from the case). **(D-F)** The concordance rate of positive expression of GC, AGG, and TLS in different regions. **(G-I)** The consistency rate of expression for GC, AGG, and TLS across different regions. (“All” represents the number of cases with consistent expression across different paraffin blocks from the same patient, while “Total” refers to the overall number of cases in the study cohort).

TLSs, including GCs and AGGs, demonstrated notable spatial heterogeneity across T, P, and N regions. **Figures 3A–C** show representative HE-stained sections from 117 ESCC patients, illustrating the detection of GC, AGG, and TLS. Assessment of different paraffin blocks from the same tumor revealed uneven distribution patterns.

The proportion of cases with complete positivity (all four blocks positive) was low in the T region (GC: 0%; AGG: 50.0%; TLS: 50.0%), but relatively higher in the P and N regions (e.g., TLS: 76.5% in P) (**Figures 3D-F**). **Figures 3G–I** further illustrate the degree of spatial consistency, defined as either all positive or all negative across blocks. GC distribution was completely consistent in 79.5% of T region samples. AGG and TLS were most stable in the P region, with consistency rates of 75.2% and 78.6%, respectively. These findings highlight the region-specific variability of TLSs and underscore the need for multi-site sampling to accurately assess the immune microenvironment in ESCC.

### Spatial distribution characteristics of TILs

**Figure 4:**
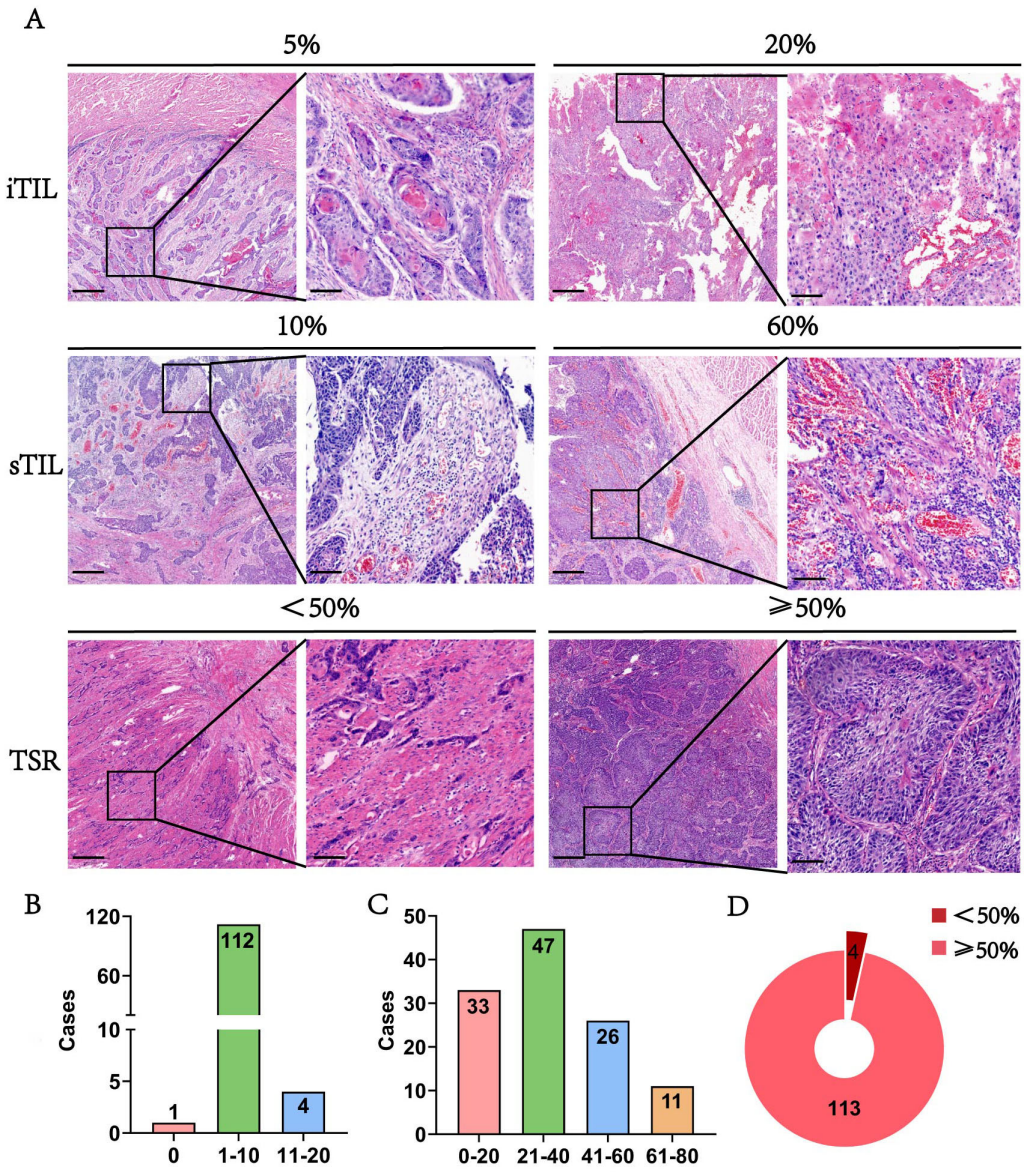
The morphology and distribution of TIL and TSR expression in the ESCC surgery cohort. **(A)** The HE morphology of iTIL, sTIL and TSR at different proportions. **(B-D)** Respectively showed the expression profiles of iTIL, sTIL, and TSR at different proportions.– – –.

In addition to analyzing TLSs, we extended our investigation to TILs to provide a more comprehensive view of immune infiltration in ESCC. TLSs offer localized information, while TILs reveal the broader spatial distribution and intensity of immune cell presence across tumor tissues. **Figure 4A** demonstrates HE staining images showing the different proportions of iTIL, sTIL, and TSR in tumor tissues. And in the ESCC surgery cohort, iTIL mostly account for 1%-10% (**Figures 4B**). sTIL are divided into four fractional regions: 0-20%, 21-40%, 41-60%, and 61-80%, with case numbers of 33 (28.2%), 47 (40.2%), 26 (22.2%), and 11 (9.4%), respectively (**Figures 4C**). TSR was ≥50% in 113 patients (96.6%), while only 4 patients (3.4%) had a TSR <50% (**Figures 4D**). These findings suggest a dominant stromal component in ESCC and indicate that iTIL infiltration is generally modest, whereas sTIL presence is more heterogeneous.

### Distribution characteristics and spatial heterogeneity of iTIL and sTIL

**Figure 5:**
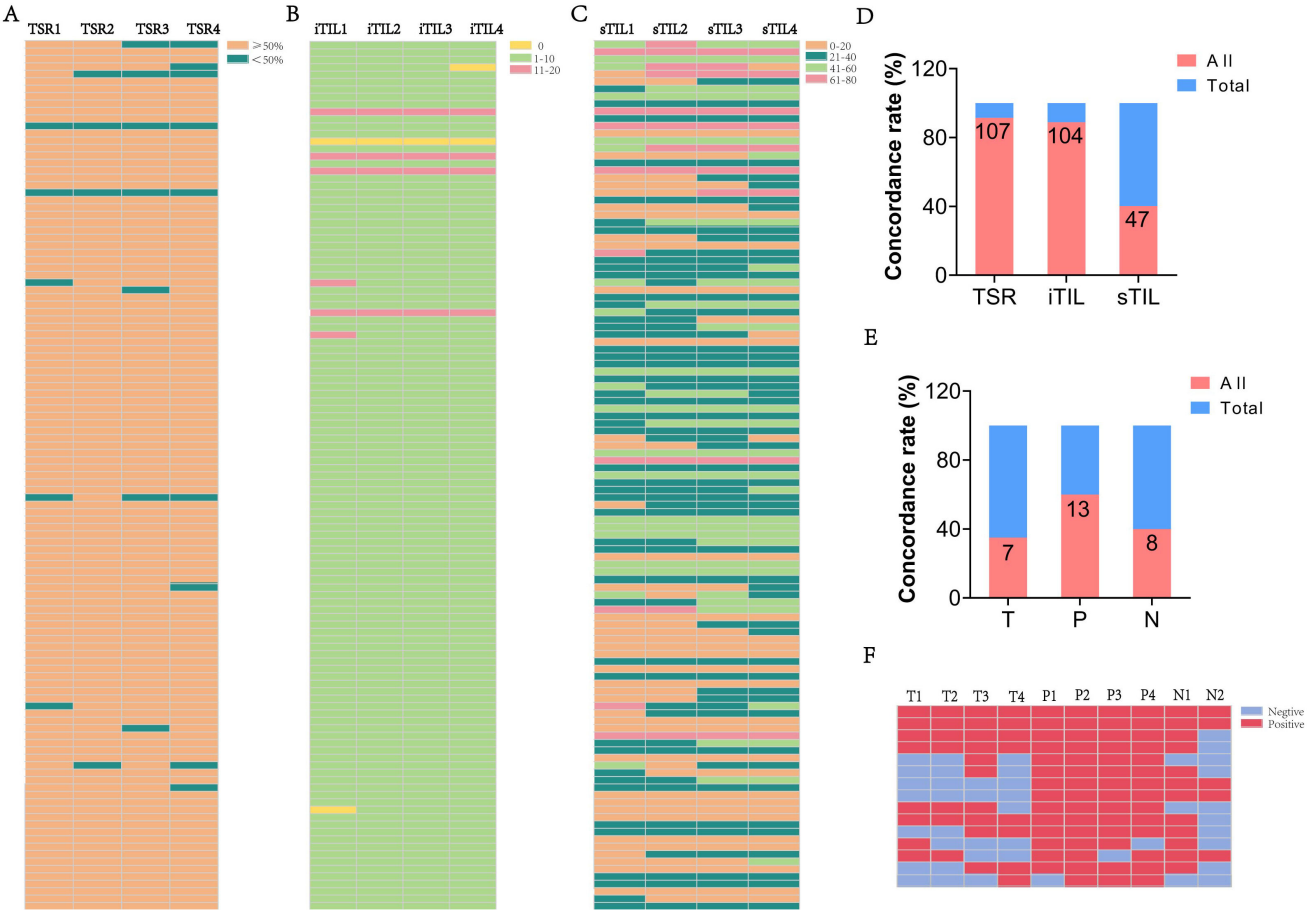
The heterogeneity of TSR and TIL in ESCC cases. **(A-C)** The proportion of TSR, iTIL, and sTIL in different paraffin blocks of each ESCC case. **(D)** The consistency rate of TSR, iTIL, and sTIL in ESCC cases. **(E)** The consistency of TLS expression in different regions T, P and N in the ESCC neoadjuvant cohort. **(F)** The expression of TLS across different paraffin blocks for each case in the ESCC neoadjuvant cohort. (“All” represents the number of cases with consistent expression across different paraffin blocks from the same patient, while “Total” refers to the overall number of cases in the study cohort).

In addition to TLSs, the spatial distribution and stability of TILs were evaluated. **Figures 5A–C** display HE staining images showing patterns of iTIL, sTIL, and TSR across multiple blocks from the surgery cohort (n = 117). Most patients exhibited a TSR ≥ 50% (88.9%) and low iTIL levels (1-10%) in 92.3% of cases. In contrast, sTIL expression showed greater variability among different tumor sections.

Consistency analysis (**Figure 5D**) revealed high spatial stability for TSR (91.5%) and iTIL (88.9%), while sTIL demonstrated significantly lower consistency (40.2%), indicating a more heterogeneous stromal immune distribution. Further insights were gained from the neoadjuvant cohort. By comparing pre-treatment biopsy samples with post-treatment surgical tissues, spatial heterogeneity was again observed. Among 15 cases with marked tumor regression (TRG 0–1), TLSs remained detectable post-treatment in 46.7% of T regions, 80.0% of P regions, and 53.3% of N regions. The consistent presence of TLSs across multiple tissue blocks in these cases (**Figures 5E–F**) reinforces the dynamic and spatially variable nature of the immune response following therapy.

### Prognostic significance of TLS with clinicopathological features

**Figure 6:**
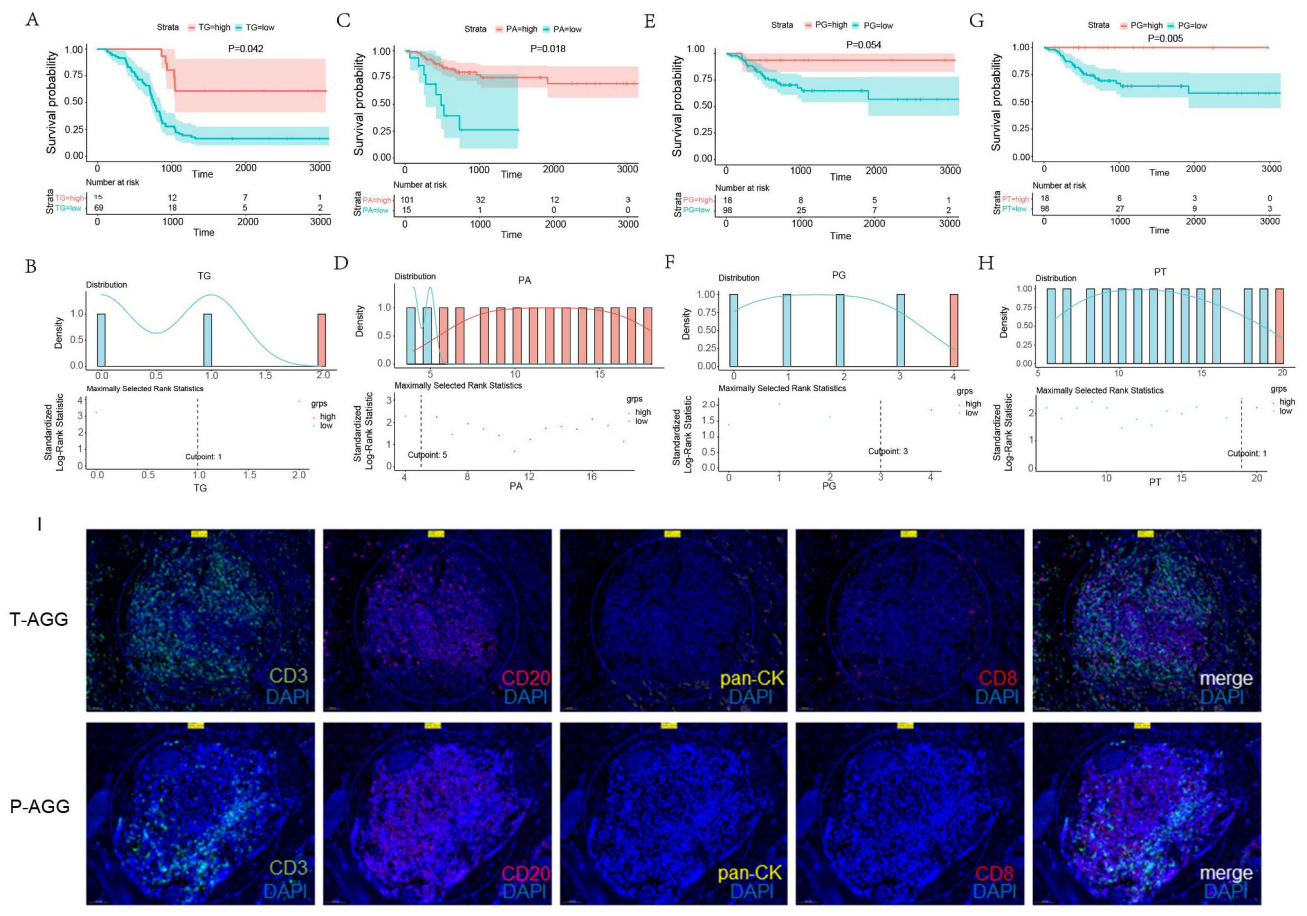
Kaplan- Meier survival curves of OS and DFS in ESCC patients, significance was assessed using the log-rank test. **(A, B)** The predictive value of GC in the tumor region for OS. **(C, D)** The predictive value of AGG in the peritumoral area for DFS. **(E, F)** The predictive value of GC in the peritumoral area for DFS. **(G, H)** The predictive value of the total TLSs in the peritumoral area for DFS. **(I)** Representative multiplex immunofluorescence staining of tumor and peritumoral regions showing CD3^+^ T cells (520), CD8^+^ T cells (620), CD20^+^ B cells (690), pan-CK^+^ tumor cells (570), and nuclei (blue, DAPI).

The presence and spatial distribution of TLSs within tumor tissues exhibit distinct prognostic implications in ESCC. KM survival analysis revealed that high presence of TG was significantly associated with longer OS in ESCC patients (log-rank *p* < 0.05) (**Figures 6A, B**). In contrast, the presence of TLSs in the peritumoral area showed no significant correlation with OS. However, high presence of perineural area (PA), grade (PG), and type (PT) was significantly associated with prolonged DFS (**Figures 6C–H**). These findings indicate that spatially resolved TLS evaluation provides valuable prognostic information beyond traditional pathological parameters. In addition, multiplex immunofluorescence staining results were consistent with the IHC findings. In the tumor region, TLS structures were characterized by aggregated CD3^+^ T cells and scattered CD20^+^ B cells. CD8^+^ cytotoxic T cells were frequently found within the CD3^+^ T-cell zones (**Figure 6I**). In the peritumoral region, TLS were segregated CD3^+^ T-cell and CD20^+^ B-cell zones, suggesting the presence of more mature TLS, including germinal center-like structures (**Figure 6I**). These findings confirm the presence and spatial organization of TLS-associated immune cells observed by IHC.

**Figure 7:**
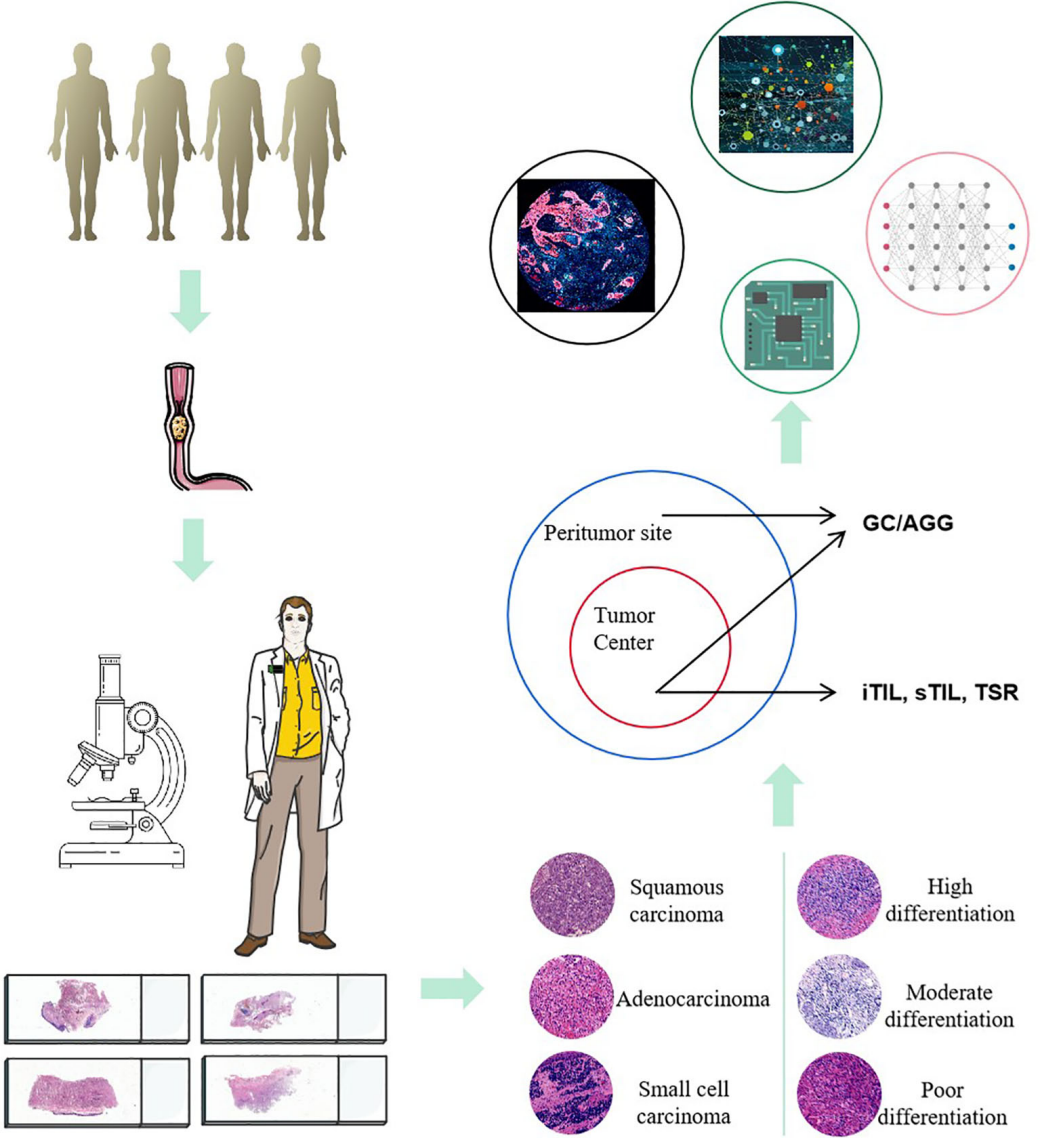
Comprehensive work plan for ESCC characterization and spatial biomarker analysis.

Building on conventional pathological diagnostics, the evaluation of TLS related indicators can provide more diagnostic and therapeutic references for the prognosis assessment of ESCC. In the future, by integrating multi-omics data (such as genomics, proteomics, and metabolomics) and predictive models using artificial intelligence, the diagnostic accuracy and treatment outcomes for ESCC can be further enhanced. This comprehensive analytical approach enables doctors to more precisely understand the biological behavior of tumors, offering more personalized and accurate treatment plans for patients (**Figure 7**).

## Discussion

This study reveals significant heterogeneity in the presence, density, and maximum diameter of TLS across different regions in patients with ESCC. This heterogeneity may influence the immune response to the tumor and its clinical prognosis. Current research indicates that the abundance and characteristics of TLS are closely related to immune responses within the tumor microenvironment (15), which may vary among different regions of esophageal cancer patients, leading to different prognostic outcomes. The presence of TLS is a favorable prognostic factor in various solid tumors (14, 16–18). Numerous studies have reported the importance of various TLS factors in prognosis, such as the number and density of TLS (19). However, this study shows that the distribution of TLS within the tumor region, adjacent tissues, and marginal tissues of the same case exhibits significant heterogeneity. In this ESCC cohort, the proportions of cases with consistent GC distribution across all paraffin blocks in the T, P, and N regions were 79.5%, 55.6%, and 60.7%, respectively; the proportions of cases with consistent AGG distribution were 53.8%, 75.2%, and 54.7%, respectively; and the proportions of cases with consistent overall TLS distribution were 54.7%, 78.6%, and 53.8%, respectively.

Tumor heterogeneity leads to different regions of the same tumor potentially possessing distinct molecular characteristics (20). The results of this study also indicate that the expression of TLS in some wax blocks or localized tissues may not reflect the overall characteristics of the patients. TLS exhibits significant heterogeneity across different tumor regions, and this heterogeneity may be related to the clonal evolution of the tumor (21). Different subclones acquire distinct molecular features during the evolutionary process (22–24). Tumor heterogeneity complicates prognosis assessment, as the biological behaviors of different subclones can vary significantly, affecting the long-term survival rates of tumor patients (25). Heterogeneity is also a major factor contributing to treatment resistance in tumors (26). Based on the heterogeneous characteristics of TLS, developing a comprehensive diagnostic strategy for the entire tumor region may help improve the precision of treatment and prognosis evaluation.

According to the multi-indicator predictive model, within the ESCC cohort, TLS and related characteristics have demonstrated prognostic value in both the surgery-only group and the surgery combined with chemotherapy group. Through this AI predictive model, this study offers pathologists a straightforward and effective method for assessing TLS characteristics in the tumors of ESCC patients. By combining microscopic and digital slide evaluations to gather TLS characteristic information, a patterned predictive procedure has been established, linking these characteristics with the prognosis and treatment response of ESCC patients, thereby supporting clinical decision-making. Furthermore, as an immune structure within the TME, the relationship between TLS and immune cells (particularly T cells and B cells) can further elucidate the mechanisms of immune evasion in ESCC (27–29). Patients with higher TLS richness may also be more likely to benefit from immunotherapy, which aids in guiding the development of individualized treatment plans (30, 31).

The advancement of immunotherapy has brought new hope for the treatment of ESCC, a disease characterized by high mortality rates and short survival times, particularly through the application of ICIs. However, not every patient benefits from immunotherapy, making the accurate screening of suitable candidates using biomarkers a significant challenge (32, 33). Our research indicates that the characteristics of TLS may offer new predictive markers for the treatment of ESCC, and the dynamic interplay between TLS and the tumor immune response highlights its potential as a critical biomarker in the field of immunotherapy. In this ESCC neoadjuvant research cohort, TLS expression was found in 62.5% of cases with TRG of 0, compared to 33.3% in cases with TRG 2-3. In the future, the molecular characteristics of TLS and their clinical applications will play a role in the precise screening of ESCC patients who are sensitive to immunotherapy (34).

This study also has certain limitations. We analyzed the relationship between TLS distribution and the survival of ESCC patients based on conventional histopathological images (including HE staining and pathological digital slides), but have not yet conducted a multi-dimensional evaluation based on new technologies. ESCC is a unique and complex heterogeneous malignant tumor (13), and artificial intelligence can be used to develop machine learning models for automated and quantitative assessment of TLS (35). In the future, we will utilize artificial intelligence (AI) and digital slides combined with more molecular indicators to further construct diagnostic and predictive models.

Multiplex immunohistochemistry (mIHC) technology is a tool for studying the tumor microenvironment (TME) (36, 37). We will utilize mIHC technology to further evaluate the clinical characteristics of molecular expression in this cohort, as well as the expression profiles of TLS-related molecules such as CD8, CD3, CD21, CD20, CD23, CD68, and PD-L1 within the TME. Additionally, we will investigate the relationship between Ki-67 expression in tumor cells and the spatial proximity of TLS to survival outcomes. In the future, we aim to further explore the relationship between TLS and tumor immune evasion mechanisms, and to investigate how modulating TLS could enhance the efficacy of immunotherapy. Moreover, with the advancement of single-cell technologies and high-throughput data analysis, in-depth research into the cellular composition of TLS and its interactions with other immune microenvironment markers will help to further unveil the potential applications of TLS (38–40). We will focus on developing new diagnostic and therapeutic strategies based on TLS characteristics to improve the survival rates and quality of life for patients with ESCC.

## Conclusions

Our results demonstrate that TLSs and TILs within the TME of ESCC exhibit significant spatial and functional heterogeneity, reflecting the complex immune landscape of ESCC. This heterogeneity may contribute to variations in treatment response and patient outcomes, highlighting the need for personalized immunotherapeutic strategies. The presence of TLSs in different regions of ESCC tissue (including GC, AGG and total TLSs) exhibit distinct clinical prognostic significance, suggesting that TLSs play a crucial role in the development and progression of ESCC. Furthermore, the interaction between TLSs and immune cells within the TME of ESCC serves as a key determinant of tumor cell fate. A deeper exploration of the underlying mechanisms will provide critical insights for the precise diagnosis and treatment of ESCC.

## Data Availability

The original contributions presented in the study are included in the article/**Supplementary Material**. Further inquiries can be directed to the corresponding author/s.
